# The MOHMQuit (Midwives and Obstetricians Helping Mothers to Quit Smoking) Trial: protocol for a stepped-wedge implementation trial to improve best practice smoking cessation support in public antenatal care services

**DOI:** 10.1186/s13012-022-01250-3

**Published:** 2022-12-09

**Authors:** Larisa Ariadne Justine Barnes, Jo Longman, Catherine Adams, Christine Paul, Lou Atkins, Billie Bonevski, Aaron Cashmore, Laura Twyman, Ross Bailie, Alison Pearce, Daniel Barker, Andrew J. Milat, Julie Dorling, Michael Nicholl, Megan Passey

**Affiliations:** 1 The University of Sydney, The University Centre for Rural Health, 61 Uralba St., Lismore, NSW 2480 Australia; 2 Northern New South Wales Local Health District, Byron Central Hospital, Ewingsdale Rd, Byron Bay, NSW 2480 Australia; 3 University of Newcastle, School of Medicine and Public Health, University Drive, Callaghan, NSW 2308 Australia; 4 University College London, Centre for Behaviour Change, Gower Street, London, WC1E 6BT UK; 5 Flinders University, College of Medicine & Public Health, Flinders Health and Medical Research Institute, Sturt Road, Bedford Park, SA 5042 Australia; 6 NSW Ministry of Health, Centre for Epidemiology and Evidence, 1 Reserve Road, St Leonards, NSW 2065 Australia; 7 The University of Sydney, Sydney School of Public Health, Faculty of Medicine and Health, Camperdown, NSW 2006 Australia; 8 Tobacco Control Unit, Cancer Prevention and Advocacy Division, Cancer Council NSW, and Conjoint Fellow, School of Medicine and Public Health, University of Newcastle, 153 Dowling St., Woolloomooloo, NSW 2011 Australia; 9 The Daffodil Centre, The University of Sydney, a joint venture with Cancer Council NSW, and Sydney School of Public Health, The University of Sydney, Edward Ford Building, A27 Fisher Rd, Camperdown, NSW 2006 Australia; 10 Western NSW Local Health District, 7 Commercial Avenue, Dubbo, NSW 2830 Australia; 11 Clinical Excellence Commission-NSW Health and The University of Sydney Faculty of Medicine and Health, 1 Reserve Road, St. Leonards, NSW 2065 Australia

**Keywords:** Implementation, Behaviour change wheel, Smoking cessation support, Pregnancy, Antenatal care, Systems change intervention, Stepped-wedge cluster-randomised controlled trial

## Abstract

**Background:**

Smoking during pregnancy is the most important preventable cause of adverse pregnancy outcomes, yet smoking cessation support (SCS) is inconsistently provided. The MOMHQUIT intervention was developed to address this evidence-practice gap, using the Behaviour Change Wheel method by mapping barriers to intervention strategies. MOHMQuit includes systems, leadership and clinician elements. This implementation trial will determine the effectiveness and cost-effectiveness of MOHMQuit in improving smoking cessation rates in pregnant women in public maternity care services in Australia; test the mechanisms of action of the intervention strategies; and examine implementation outcomes.

**Methods:**

A stepped-wedge cluster-randomised design will be used. Implementation of MOHMQuit will include reinforcing leadership investment in SCS as a clinical priority, strengthening maternity care clinicians’ knowledge, skills, confidence and attitudes towards the provision of SCS, and clinicians’ documentation of guideline-recommended SCS provided during antenatal care. Approximately, 4000 women who report smoking during pregnancy will be recruited across nine sites. The intervention and its implementation will be evaluated using a mixed methods approach. The primary outcome will be 7-day point prevalence abstinence at the end of pregnancy, among pregnant smokers, verified by salivary cotinine testing. Continuous data collection from electronic medical records and telephone interviews with postpartum women will occur throughout 32 months of the trial to assess changes in cessation rates reported by women, and SCS documented by clinicians and reported by women. Data collection to assess changes in clinicians’ knowledge, skills, confidence and attitudes will occur prior to and immediately after the intervention at each site, and again 6 months later. Questionnaires at 3 months following the intervention, and semi-structured interviews at 6 months with maternity service leaders will explore leaders’ perceptions of acceptability, adoption, appropriateness, feasibility, adaptations and fidelity of delivery of the MOHMQuit intervention. Structural equation modelling will examine causal linkages between the strategies, mediators and outcomes. Cost-effectiveness analyses will also be undertaken.

**Discussion:**

This study will provide evidence of the effectiveness of a multi-level implementation intervention to support policy decisions; and evidence regarding mechanisms of action of the intervention strategies (how the strategies effected outcomes) to support further theoretical developments in implementation science.

**Trial registration:**

ACTRN12622000167763, registered February 2nd 2022.

**Supplementary Information:**

The online version contains supplementary material available at 10.1186/s13012-022-01250-3.

Contributions to the literature

- MOHMQuit, an evidence-based systems-change intervention, will be implemented at nine public maternity services using existing health care capacity.
- This trial explicitly tests a theory and framework-driven approach relative to many earlier interventions which were less clearly built on implementation science frameworks and will provide further empirical evidence of the effectiveness of this approach.
- A comprehensive suite of measures will examine effectiveness and cost-effectiveness; and test mechanisms of action of the intervention strategies and factors impacting implementation to advance the field of implementation science.

## Background

Smoking during pregnancy is the most important preventable cause of adverse pregnancy outcomes, maternal-foetal health complications and ongoing developmental complications in the infant [1, 2]. Smoking during pregnancy doubles or triples the risk of multiple complications including stillbirth, preterm birth and low birth weight and birth defects and sudden infant death syndrome [1, 3–5]. Smoking also increases the health risks to the mother, including her risk of developing cancer, coronary heart disease and stroke; all of these are significantly reduced if women stop smoking during pregnancy [2]. Quitting smoking at any stage of pregnancy is beneficial to both mother and baby, and quitting in the first half of pregnancy reduces the risk of preterm birth and small for gestational age babies to that of non-smokers [3]. Reducing smoking rates in pregnancy is a key priority in health systems around the world to reduce the increased morbidity and mortality in mothers and babies and optimise children’s development in the first 2000 days [6–9].

In 2019, 9.0% of pregnant women in Australia smoked in the first half of pregnancy, with most (75%) continuing to smoke in the second half [10]. Higher smoking rates are found in pregnant women who live in remote areas of Australia, are of low socio-economic status, of Aboriginal or Torres Strait Islander identity or are teenage mothers [11–13]. As such, smoking in pregnancy is considered a major public health concern and supporting women to quit smoking in pregnancy can help reduce the health inequities faced by these women and their children [6, 14]. Although many pregnant women are highly motivated to quit, they face significant challenges including lack of consistent, effective support from health professionals [15, 16], despite evidence that shows that when clinicians offer consistent smoking cessation support (SCS) using psychosocial interventions it helps pregnant women to quit [17]. Pooled data from a Cochrane systematic review also found that among women who received these interventions there was a 17% reduction of infants born with low birthweights and a 22% reduction in neonatal intensive care admissions [17].

Evidence-based Australian guidelines [14, 18] recommend routine SCS be delivered during antenatal care for all pregnant women using brief interventions based on the 5As (Ask, Advise, Assess, Assist and Arrange follow-up) and include providing nicotine replacement therapy (NRT) if women are otherwise unable to quit. However, provision of recommended SCS to pregnant women has remained persistently poor [19–21]. A state-wide survey of women’s experiences of maternity care in New South Wales (NSW) found that only 46% of women who smoked in pregnancy recalled being told about quitting programs [22]. Midwives, obstetricians and managers all reported major gaps in care, particularly in assisting women with cessation strategies and arranging follow-up [23, 24], both of which are crucial to quitting success [25, 26]. Despite the availability of guidelines and training, maternity care services have faced considerable barriers in implementing SCS [23, 27] illustrating the persistence of an evidence to practice gap. One possible reason for the gap has been lack of comprehensive and theoretically informed interventions to support services in delivering SCS, suggesting a theoretically underpinned systems change approach to guideline implementation is needed [28, 29].

Systems change interventions in public health services have been shown to be effective in increasing identification of smokers by clinicians and documentation of smoking status in electronic medical records, improving integration of SCS into usual care, and increasing the numbers of referrals to telephone Quitline services [30–32]. However, to date, only one adequately powered trial has tested the effectiveness of theoretically informed implementation strategies in antenatal settings [33].

In addition to the need to select implementation strategies based on theory with a clear specification of the interventions [34], there have been recent calls for more rigorous testing of *how* implementation strategies work (or do not)—the mechanisms of action of the implementation strategies [35, 36]. To achieve this, implementation strategies need to be based on a sound understanding of the barriers to implementation and clearly explicate how the strategies are intended to address the identified barriers, thus generating testable hypotheses to assess if the strategy worked—or how multiple strategies may work together to achieve the desired outcome [35, 36]. This approach will assist to advance the scientific basis of implementation research.

## Methods

### The implementation intervention-MOHMQuit

To better support clinicians in helping women to stop smoking in pregnancy we developed a systems-change intervention (MOHMQuit: Midwives and Obstetricians Helping Mothers to Quit), using a theoretically underpinned intervention development process, the Behaviour Change Wheel [37]. The process of developing the MOHMQuit intervention is described in depth elsewhere [15]. Briefly, we used the Theoretical Domains Framework—a framework of psychological constructs in behaviour change theory [38]—to identify barriers and enablers clinicians faced to providing evidence-based SCS during antenatal care. We undertook qualitative research with maternity service managers, midwives and obstetricians [23] and a state-wide cross-sectional anonymous survey of midwives working in antenatal care [24]. Working closely with key stakeholders in the NSW public health system we then applied the steps of the Behaviour Change Wheel method [37] to develop the initial MOHMQuit intervention. This was followed by a trial at one site to determine the feasibility and acceptability of the intervention with maternity service leaders and midwives [15]. Following further refinements, the implementation, effectiveness and cost-effectiveness of the MOHMQuit intervention is now being tested. MOHMQuit is a multi-strategy systems-change intervention designed to be sustainable, which addresses identified barriers and enablers for clinicians providing SCS and ensures ongoing support for SCS amongst service managers.

The intervention includes multiple strategies with systems, leadership and clinician elements (see Additional file 1 which maps the intervention types and behaviour change techniques to the previously identified barriers). The systems change elements include building leadership capacity, improving recording of SCS in *e*Maternity (the electronic health record used by maternity services in NSW), and various resources for both leaders and clinicians, as described below. All maternity service leaders, clinicians, and Aboriginal health workers will be asked to complete two NSW Health Education and Training Institute (HETI) [39] online modules to ensure basic knowledge of smoking cessation prior to MOHMQuit-specific training. (See Table 1 for definitions of maternity service leaders, clinicians and Aboriginal health workers used in this research.) MOHMQuit includes provision of a range of resources with a series of workshops held at each site to train and support participants in their use. All workshops use evidence-based behaviour change techniques (e.g. social comparison, modelling, behavioural practice/rehearsal, reframing smoking) [37, 40, 41]. To maximise the sustainability of the intervention, midwifery educators will be trained in providing ongoing MOHMQuit training, and sites will be supported by the development of a ‘Community of practice’ to provide additional and ongoing peer support and encouragement. Additional details of the delivery of the intervention are given below and in Additional file 2.

**Table 1:** Maternity service leaders, clinicians, and Aboriginal health workers

Personnel	Role/s in maternity services relevant to MOHMQuit
Maternity service leaders	Defined as maternity service leaders who support or supervise clinicians providing antenatal care at each site, its catchment and associated services. These include clinical midwifery consultants, maternity unit managers, clinical midwifery educators, clinical midwifery specialists, clinic coordinators, obstetric leads and others in leadership positions (these may vary slightly by site).
Clinical midwifery educators	Clinical midwifery educators are experienced midwives who undertake additional roles to maintain and advance the clinical practices of maternity care clinicians, working within professional development frameworks to support ongoing education [42, 43]. Midwifery educators play crucial roles in the quality and safety advancement of health services, helping to ensure safe practices are maintained and required clinical competencies are achieved [42].
Maternity care clinicians	All midwives, obstetricians and obstetric trainees providing antenatal care.
Aboriginal health workers	All Aboriginal health workers who provide antenatal care. Aboriginal health workers are primary health care workers who provide clinical and primary health care, supporting women independently or with other maternity care clinicians to ensure the provision of culturally safe maternity care [44, 45].

#### Maternity service leaders’ workshop

The 3-h maternity service leaders’ workshop is designed to enhance leadership in supporting clinicians to provide SCS. The workshop will be conducted by a senior midwifery trainer. Leaders will be provided with a range of resources and supported to use them to enhance service delivery. On workshop completion, leaders will be asked to: encourage and support clinical staff to attend the relevant training (see below); complete an action planning tool and review annually; identify, develop and maintain a local smoking cessation support champion; develop local care pathways for smokers; and other actions guided by their action plan. Additionally, leaders will be asked to review *e*Maternity reports monthly and discuss with their team as part of an audit and feedback process to improve professional practice [46] and help contribute to the effective implementation of the intervention [47].

#### Clinician workshops

Training for clinicians will be jointly provided by a midwifery trainer and a smoking cessation training expert. The workshops will demonstrate use of a suite of resources, with opportunity to practise their use, with the aim of building confidence and skills in supporting smoking cessation. Training for midwives and Aboriginal health workers will be provided in a 1-day workshop, while training of approximately 2 h will be provided for obstetricians and obstetric trainees. As an incentive for participation, continuing professional development points will be awarded for all clinicians.

#### Clinical midwifery educator training

Clinical midwifery educators will be provided training and resources to continue to deliver training following the intervention phase, to address issues of staff turnover or absence, in order to maximise sustainability of the intervention. Clinical midwifery educators will attend the 1-day training with the midwives and Aboriginal health workers, and then an additional 1 h training on how to provide the training themselves.

#### Development of a community of practice

Sites will be encouraged to participate in an online ‘Community of practice’ to provide additional and ongoing peer support and encouragement. Senior members of the research team will also provide ongoing support for the implementation of MOHMQuit across all sites at these monthly meetings. Each site will be added to the Community of practice meetings after training occurs at their site.

**Fig. 1:**
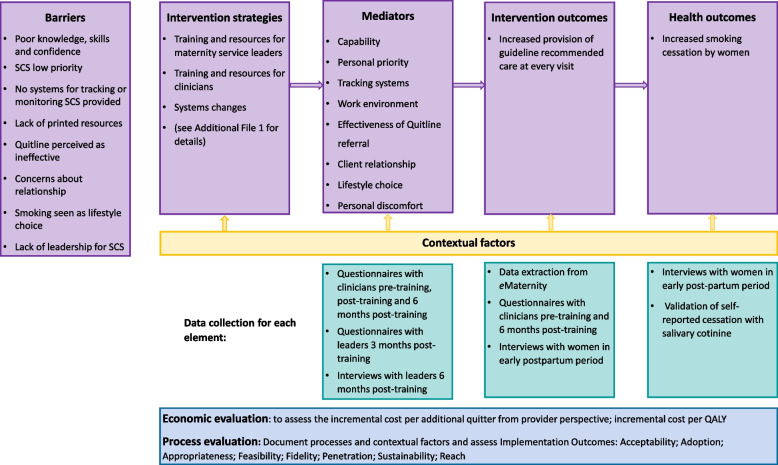
MOHMQuit implementation logic model

The implementation logic model, with data collection to evaluate it, is shown in Fig. 1.

### Study design

The study is being undertaken as a partnership between academic researchers, policy makers within NSW State Government and non-government agencies and senior clinicians from participating sites. At each site, there is a Midwifery Partner Investigator and an Obstetric Partner Investigator supporting implementation of the trial.

The MOHMQuit trial is a pragmatic stepped-wedge cluster-randomised controlled trial of an implementation intervention in nine public maternity services in NSW. Our overarching goal is to increase smoking cessation among pregnant women to improve health outcomes. This trial will test the effectiveness and cost-effectiveness of the MOHMQuit intervention in achieving this, while also testing the mechanisms of action of the strategies based on the underpinning theoretical development of MOHMQuit. The specific aims of the trial are to compare the effectiveness of MOHMQuit versus usual care in increasing:

1. Smoking cessation among pregnant women attending public antenatal services in NSW;
2. The provision of guideline-recommended SCS as documented in *e*Maternity;
3. Participating clinicians’ self-reported provision of guideline-recommended SCS to pregnant women;
4. Participating clinicians’ knowledge, skills, confidence and positive attitudes regarding providing guideline-recommended SCS to pregnant women; and
5. Women’s reported receipt of cessation advice, resources and referral to quit smoking services.

Additional aims include the following:

6. Determining the cost-effectiveness of the intervention in increasing smoking cessation;
7. Assessing implementation of the intervention through a detailed process evaluation;
8. Examining the mechanisms of action of the intervention strategies and moderators of their impact.

### Primary hypothesis

Among pregnant smokers (women reporting current smoking at antenatal booking) there will be a 5% increase in cotinine-confirmed 7-day point prevalence abstinence from a baseline prevalence of 16%, after introduction of MOHMQuit. This conservative estimate is based on the effectiveness of antenatal psychosocial smoking cessation interventions in the latest Cochrane review (RR 1.44, 95%CI 1.19-1.73) [17].

### Settings

The MOHMQuit trial will be implemented in nine public maternity services in NSW. The sites vary in size and are located in rural and urban contexts.

### Stepped-wedge cluster-randomised controlled trial design

**Fig. 2:**
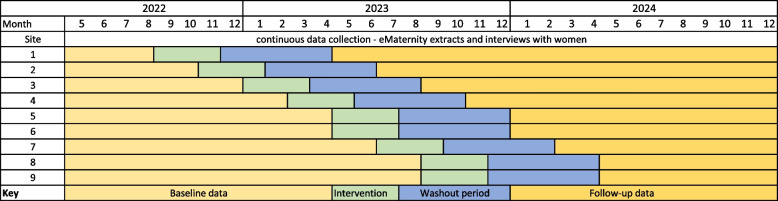
Stepped-wedge design of the MOHMQuit trial

The MOHMQuit trial uses a stepped-wedge cluster-randomised controlled trial design with random allocation of services to the intervention order [48–50]. All services begin as part of the control condition and are randomised to transition from the baseline condition (standard care) to the intervention at specific intervals or ‘steps’ [48, 49]. Each step will occur at two monthly intervals (Fig. 2). There is a 3-month intervention period during which the intervention is provided and embedded in the service, and a 5-month ‘washout period’ to allow time for women receiving care in the baseline period to complete their pregnancies, thus eliminating contamination between baseline and follow-up periods (Fig. 2). The trial was originally planned to run over 36 months at eight sites. However, due to the impacts of COVID-19 and some redesign work being completed in *e*Maternity, initiation of data collection and provision of the intervention was delayed. To compensate for the reduced time available for data collection, an additional site was added (site six in Fig. 2), and the timing of providing the intervention at the final site brought forward. This ensured the study was still adequately powered.

The stepped-wedge cluster-randomised controlled trial design was chosen because the intervention is at the service level and this design is recommended as a “pragmatic randomised study design… for the evaluation of service delivery outcomes” ([49], p6). Additionally, a stepped-wedge design is more powerful than a parallel cluster design in situations where there are relatively small numbers of eligible services; and it allows sites to act as their own historical controls, while also allowing observation of any within site temporal trends [49, 51]. While it is possible to offer the intervention to control sites at the end of a parallel cluster design trial, there are rarely sufficient time or resources remaining to provide this effectively and ethically. Additionally, the level of evidence provided by a stepped-wedge design is considered to be robust and comparable with other cluster randomised controlled trial designs [48, 49].

To prepare the MOHMQuit study protocol paper we have followed the Consolidated Standards of Reporting Trials (CONSORT) extension for the stepped wedge cluster randomised trial [50] (Additional file 3) and where applicable, the TIDieR (Template for Intervention Description and Replication) Checklist [52] (Additional file 4).

### Participants

#### Eligibility criteria—study sites

To be eligible for participation, maternity services were required to provide antenatal care and birthing services and to also (i) have a prevalence of smoking in the first half of pregnancy ≥ 12%; and (ii) have a minimum of 80 women smoking in the first half of pregnancy per year [53]. These criteria ensured that the intervention will be offered in services with smoking prevalence higher than the Australian average of 9.5% in the first half of pregnancy [11]; and provide an adequate number of smokers at each site. All antenatal services providing care to women intending to give birth at these sites were eligible to participate, including hospital-based, community-based and outreach services. Nine eligible maternity services have confirmed participation.

#### Eligibility: participants—maternity service leaders and clinicians

The following clinicians working in public maternity care services at participating sites will be eligible to participate:

- *Maternity service leaders (leaders):* all maternity service leaders who support or supervise clinicians providing antenatal care at each site, its catchment, and associated services, will be eligible (see Table 1).
- *Maternity care clinicians (clinicians):* all clinicians providing antenatal care, including midwives, obstetricians, obstetric trainees, and Aboriginal health workers, will be eligible to participate (see Table 1).

#### Eligibility: participants—pregnant and postpartum women

Pregnant/postpartum women who meet all the criteria below will be eligible to participate:

- Received antenatal care through participating maternity services (including community based and outreach services and the Aboriginal Maternal and Infant Health Service [44])
- Birthed their baby at one of the participating services during the study period
- Indicated that they were smokers or quit during this pregnancy at their first antenatal appointment with a health care practitioner at the participating maternity services, as recorded in eMaternity. (Smoking status is a required field in eMaternity at this initial visit).

Pregnant women receiving *antenatal* care at participating sites will be excluded from the study if:

- They indicate they wish to opt-out from the study
- They experience a perinatal death in this pregnancy or birth
- Their baby is transferred to a Neonatal Intensive Care Unit
- They do not speak English
- They are less than 16 years of age at the time of data collection
- A Research Midwife assesses that they are unable to provide informed consent

### Recruitment and consent

#### Services

Prior to the submission of the trial funding application, maternity services across NSW meeting these criteria were approached to explore interest in participation*—*9 of 15 services approached agreed to participate and contributed to the funding application.

#### Staff (maternity service leaders and clinicians)

##### Maternity service leaders

The study will be promoted by the Midwifery Partner Investigators at each site. While each site has already agreed to participate in the trial, individual maternity service leaders and clinicians will still be provided with a Participant Information Statement (PIS) and asked to provide individual consent to participate in data collection processes. They will be invited to participate by their manager and be provided with detailed information about the study. Written consent to participate will be sought at the leaders’ workshop.

##### Maternity care clinicians

All clinicians providing antenatal care will be informed of the trial by their manager or other service leaders, provided with detailed information and invited to participate in the trial and provide consent to the data collection processes. This process is consistent with usual practice in health services and with our pragmatic study design.

#### Pregnant and postpartum women

A two-stage recruitment and consent process will be used to recruit pregnant and postpartum women to the study. In an initial opt-out process, all pregnant women presenting for antenatal care at the services will receive a PIS and be given the option of opting out of the study by contacting a research midwife by text. For women who remain and meet the eligibility criteria, a research midwife will contact them by text and then a telephone call, give a verbal explanation of the study, encourage women to ask any questions, and ask women to provide verbal consent to participating in a brief telephone interview. Women who consent to the telephone interview, and who report smoking abstinence in the 7 days before the birth of their baby, and since giving birth, will also be asked to consent to giving a saliva sample for cotinine testing. Importantly, all women will receive full ante- and postpartum care regardless of whether they consent to participate in the study.

### Data collection and analysis

Mixed methods evaluation of the intervention and its implementation will be undertaken. Mixed methods evaluations are recommended as being practical and suitable for implementation research [54, 55]. Using a mixed methods evaluation will enable the multiple perspectives of leaders, clinicians and pregnant women to be understood, while examining multiple types of outcomes [54].

**Fig. 3:**
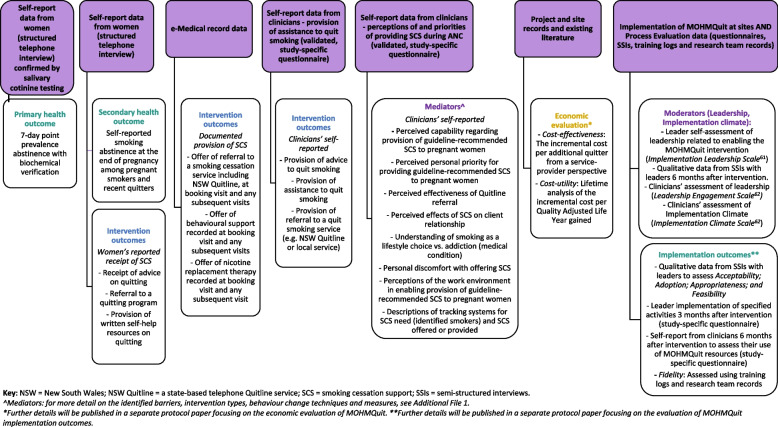
MOHMQuit data sources and outcomes

Data on the intervention and health outcomes and the implementation process will be collected by the research team, independently from each site. Variables to be collected, with the method of collection and source, timepoint of measurement and methods of analysis are shown in Table 2. Information on the types of data collected from each source are also illustrated in Fig. 3. An explanation of the relationship between the identified barriers, intervention types, behaviour change techniques and measures for the mediators are shown in Additional file 1. Analysis for aim 8 (examining the mechanisms of action of the intervention strategies and moderators of their impact) which assesses the interactions between many of the variables, is provided below Table 2. More detail regarding the process evaluation and the economic evaluation will be provided in separate protocol papers.

**Table 2:** MOHMQuit data collection and analysis

Measure	Methods of data collection	Source	Timepoint	Data analysis
**Primary health outcome**				
Biochemically verified 7-day point prevalence abstinence at the end of pregnancy among pregnant smokers and recent quitters, confirmed by salivary cotinine testing. (Aim 1)	Structured telephone interview^a^ and salivary cotinine testing	Postpartum women	Within 2 weeks after giving birth	• The primary analysis will compare the odds of quitting smoking (cotinine-confirmed 7-day point prevalence abstinence) after the adoption of the intervention relative to the baseline rate. • To achieve this, logistic regression will be used within a generalised linear mixed model framework. • In addition to the fixed effect for intervention, a fixed effect for time will be included to adjust for any secular trends in the outcome. Random effects for site and time within site will be included to account for clustering by site and repeated measures over time. • Women who do not complete the biochemical verification will be classified as continuing smokers, as recommended in analyses of smoking cessation trials (e.g., see [56]).
**Secondary health outcome**				
Self-reported 7-day point prevalence abstinence at the end of pregnancy among pregnant smokers and recent quitters. (Aim 1)	Structured telephone interview^a^	Postpartum women	Within 2 weeks after giving birth	Self-reported 7-day point prevalence abstinence will be analysed in the same way as the primary outcome.
**Intervention outcomes**				
Documented provision of smoking cessation support (SCS) at booking visit and any subsequent visits. Offer of: • referral to a smoking cessation service including NSW Quitline • behavioural support • nicotine replacement therapy (Aim 2)	Deidentified data extraction of specified variables for all births to eligible women at intervention sites	*e*Maternity^b^	Throughout the 32-month collection period	• *e*Maternity SCS scores will be calculated based on the documented provision of SCS in *e*Maternity for each woman at each visit. • Scores will be modelled using a linear mixed model. Fixed effects will be included for time, intervention, and the interaction of these. •Random effects for site and participant will be included to account for clustering by site, and repeated measures on participants. •The interaction term will directly compare the rate of change in SCS scores over antenatal visits between pre and post-intervention.
Self-reported: • Provision of Advice to quit smoking • Provision of Assistance to quit smoking • Provision of referral to a quit smoking service (e.g. NSW Quitline or local service). (Aim 3)	Study specific questionnaire	Clinicians	Before training and 6 months post-training	• The outcome measure will be a composite score of self-reported provision of SCS, calculated from the questionnaires completed by clinicians prior to training and at 6 months post-training. • Post-intervention clinician SCS scores will be modelled using a linear mixed model. Fixed effects will be included in the model for the pre-intervention SCS score and time. A random effect will be included for site to account for randomisation by cluster.
Women’s reported receipt of: • Advice on quitting • Referral to a quitting program • Written self-help materials (Aim 5)	Structured telephone interview^a^	Postpartum women	Within 2 weeks after giving birth	• Receipt of SCS reported by women will be modelled using logistic regression in a generalised linear mixed model framework. Fixed effects for time and intervention will be included in the model and a random effect for site will be used to account for randomisation by cluster.
**Mediators** (for more detail on the identified barrier, intervention types, behaviour change techniques and measures, see Additional file 1)				
• Capability • Personal priority • Tracking systems • Work environment • Effectiveness of Quitline referral • Client relationship • Lifestyle choice • Personal discomfort (Aim 4)	Study-specific questionnaire	Clinicians	Before training, immediately post-training and 6 months post-training	• Factor analysis of the initial questionnaire used to identify barriers/enablers identified several factors that predicted provision of SCS (e.g. capability, personal priority). These are included in the current questionnaire with clinicians. • Additional items to measure constructs/barriers identified in our qualitative research, but not in the original questionnaire have been added to enable assessment of whether these barriers have been addressed. • Initially, confirmatory factor analysis will be undertaken to confirm the factor structure. • A composite score across all items within each of the factors will be the outcome measure. • Any items not loading onto a factor will be modelled separately. • To assess changes in factor scores the total score on each factor will be modelled using a linear mixed model where one parameter will estimate the score immediately post-intervention and another parameter will estimate the score 6 months after intervention. The model will adjust for pre-intervention total score and time. A random effect will be included for site to account for randomisation by cluster. • Note - work environment will only be assessed immediately pre-training and 6 months post-training, as this is unlikely to have changed during the training.
**Moderators**				
Leadership (Aim 8)	Study specific questionnaire includes the *Implementation Leadership Scale* [57]	All leaders involved in training	Three months after leader training	• The Implementation Leadership Scale (supervisor version) is comprised of twelve items that make four subscales: the proactive subscale, the knowledgeable subscale, the supportive subscale, and the perseverant subscale. • For each subscale, a mean score for each set of items that load onto the relevant subscale will be computed. Additionally, a mean of the scale scores will be computed to generate the total score for the Implementation Leadership Scale [57]. Scores will be computed for each clinician and an aggregated clinical level score will be calculated.
	Study specific questionnaire includes the *Leadership Engagement Scale* [58]	Clinicians	6 months post-training	The Leadership Engagement Scale is comprised of four items. A mean score for these items will be computed for each clinician. An aggregated clinic level score will also be calculated.
	Interviews	Selected leaders	6 months post-training	Analysis of qualitative data is described below under process evaluation.
Implementation climate (Aim 8)	Study-specific questionnaire includes the *Implementation Climate Scale* [58]	Clinicians	6 months post-training	The Implementation Climate Scale is comprised of four items. A mean score for these items will be computed for each clinician. An aggregated clinic level score will also be calculated.
**Implementation outcomes**				
Acceptability (Aim 7)	Interviews	Selected leaders	6 months post-training	Qualitative data will be analysed thematically [59] to understand perceptions of acceptability, variation in this, and factors impacting it.
Adoption (Aim 7)	Study specific questionnaire	Clinicians	6 months post-training	At each site the proportion of respondents reporting utilisation of each of the key resources will be calculated.
	Study specific questionnaire	Leaders	3 months post-training	For each site, the reported completion of each of the leader components will be calculated.
	Interviews	Selected leaders	6 months post-training	Qualitative data will be analysed thematically [59] to understand the extent of implementation undertaken and factors impacting this.
Appropriateness (Aim 7)	Interviews	Selected leaders	6 months post-training	Qualitative data will be analysed thematically [59] to understand perceptions of the appropriateness of the intervention, and any adaptations made.
Feasibility (Aim 7)	Interviews	Selected leaders	6 months post-training	Qualitative data will be analysed thematically [59] to understand perceptions of the feasibility of implementing MOHMQuit and the extent to which it has been integrated into usual care.
Fidelity (of delivery of the MOHMQuit intervention to the services) (Aim 7)	Training logs Researcher notes	Research team	Throughout	Data will be analysed to determine the proportion of eligible clinicians and leaders participating in training; the extent to which the training and other strategies were delivered as intended.
Context	Multiple	Internet, Partner Investigators and Leaders	Throughout	Information on service size, capacity, structure will be collated. Information from Partner Investigators and leaders regarding factors impacting delivery will be documented throughout the study period.
**Economic evaluation**				
Cost-effectiveness—the incremental cost per additional quitter from a service-provider perspective (Aim 6)	Within-trial data collection	Data will be sourced from study and site records as well as the existing literature.	Data collection throughout the study, with analysis at the end.	Economic evaluation conducted from the perspective of the Australian health care system with 8-month time horizon for cost-effectiveness and lifetime time horizon for cost utility. Probabilistic sensitivity analysis and non-parametric bootstrapping will be used to assess robustness of the model.
Cost-utility—lifetime analysis of the incremental cost per Quality adjusted life year (QALY) gained (Aim 6)	Modelled economic evaluation	Data will be sourced from study and site records as well as the existing literature.	Data collection throughout the study, with analysis at the end.	

*Key: NSW* New South Wales, *NSW Quitline* A state-based telephone Quitline service, *SCS* smoking cessation support, *eMaternity* the electronic health record used by maternity services in NSW.

^a^Study specific interview with postpartum women: women will be interviewed by a research midwife by telephone within 2 weeks of giving birth and asked about their smoking status in the final 7 days of their pregnancy. They will also be asked their perspectives of SCS provided in antenatal care using a structured interview schedule.

^b^Data extractions from eMaternity: deidentified, quantitative data will be extracted from the eMaternity database throughout the 32-month data collection period to analyse clinicians’ provision of best-practice SCS as documented in electronic-medical records (aim 2).

#### Primary outcome (health outcome)

A 7-day point prevalence abstinence is an established abstinence outcome and can be combined with biochemical verification [56, 60]. For women who report at least 7 days of abstinence at the end of their pregnancy, and continued abstinence since, home-visiting midwives will collect saliva samples which will be tested for cotinine levels at a central pathology facility. The samples will be tested using liquid chromatography with tandem mass-spectrometry and a cut-point of 8 μg/L. A 7-day point prevalence abstinence with biochemical verification is commonly used in pregnancy smoking cessation trials, as longer timeframes (e.g. 12-month abstinence) are not relevant to benefits to the foetus [61, 62]. As biochemical verification will not always be possible, self-reported 7-day point prevalence (regardless of verification) will be used as a secondary outcome.

### Analysis to examine the mechanisms of action (aim 8)

To examine the causal linkages between the strategies, mediators, moderators and implementation outcomes structural equation modelling will be undertaken using data from clinician surveys collected pre-training and 6 months post-training. The primary outcome will be the change in the composite score of self-reported provision of SCS between pre-training and 6 months post-training. We will use structural equation modelling to estimate the direct and indirect effects of each of the intervention strategies (as articulated in Additional file 1) mediated by changes in the mediator scores listed in Table 2. A detailed statistical analysis plan will be developed.

### Sample size

The trial aims to recruit 4320 pregnant women who smoke over the 32 months of data collection across the nine sites.

NSW Health data indicate there were over 2000 women smoking in the first half of pregnancy in the participating sites in 2018. Assuming that at each of the 9 sites on average 40 participants will consent every 2 months, the total sample size available will be 9 × 40 × 12 = 4320. Using a significance level of 5% and assuming an intra cluster correlation (ICC) of 0.02 and a cluster autocorrelation of 0.7, the primary analysis will have more than 80% power to detect an increase in 7-day point prevalence abstinence of 5% from a baseline prevalence of 16% (i.e. intervention 21% vs control 16%). This conservative estimate is based on the effectiveness of antenatal psychosocial smoking cessation interventions in the latest Cochrane review (RR 1.44, 95%CI 1.19–1.73) [17].

### Randomisation and blinding

The nine services have been randomly allocated to each ‘step’ (Fig. 2) by an independent statistician using a computerised simple random selection without replacement regime. This ensures no service is unjustifiably favoured by receiving the intervention before another service. Randomisation occurred after all clusters agreed to participate to reduce bias arising from the randomisation process and thus increase the internal validity of the study [51].

Pregnant women receiving antenatal care, the research midwives interviewing the women, and staff analysing the data will be blinded to the intervention phase. It is not possible to blind maternity service leaders and clinicians at participating sites to the intervention phase. However, risk of bias for the primary aim is reduced as smoking status will be biochemically verified. The regular data extraction of SCS recorded in *e*Maternity combined with the data from the interviews with women of their recall of SCS received, may mitigate these potential biases.

## Discussion

### ‘Real world’ evidence and contribution to implementation science theory

By providing a robust, comprehensive examination of whether our multi-strategy intervention improves SCS in maternity care and cessation among women, the MOHMQuit trial will deliver ‘real world’ evidence of the effectiveness and cost-effectiveness of the MOHMQuit intervention. The thoroughness and theoretical underpinnings of the design [15, 37, 38], feasibility and pilot investigations [15], with a clearly articulated logic model, are strengths of this trial that will also enable testing the impact of the intervention on the hypothesised mediators and testing the mechanisms of action of the intervention strategies, contributing empirical evidence to the theoretical foundations of implementation science [35, 36]. The MOHMQuit trial will provide important evidence for systems change interventions that include three levels of influence (systems, leaders and clinicians) to instigate behaviour change in clinicians, improve the provision of SCS, and ultimately reduce smoking rates in pregnancy.

Data collection has been specifically designed to test whether the MOHMQuit intervention has addressed the barriers to provision of SCS identified in our previous research, and which the intervention was carefully designed to address [15, 23, 24, 63]. Use of previously validated data collection instruments will allow us to assess this [24]. Additionally, the process evaluation (described in more detail in a separate paper) will assess implementation outcomes and adaptations, adding further understanding regarding how to implement evidence-based practice in real world settings, while adding to the empirical evidence to support further theoretical developments in implementation science.

### Advantages of the pragmatic stepped wedge trial design

The MOHMQuit trial has been designed as a pragmatic trial according to the PRECIS-2 criteria [64]. The stepped-wedge cluster-randomised design is a pragmatic study design commonly used in the evaluation of service delivery interventions [50]. Policy makers need strong evidence of effectiveness and cost-effectiveness in ‘real world’ conditions [65]. A strength of this design is that it focuses on carrying out ‘real-world’ research, which in this case is representative of genuine clinical practice in the provision of maternity care in public antenatal services with a range of different models of care [66]. This trial will also provide evidence of the practicality of using the MOHMQuit intervention in the provision of ‘real world’ maternity care provided by usual antenatal care providers, with all pregnant women who smoke or quit smoking since becoming pregnant being eligible to participate [66].

The stepped-wedge design has several advantages for translational research, including that (a) it controls for between-service variation in baseline practice; (b) statistical power is boosted as it allows assessment of intervention effects in a pre-/post-comparison across services; and (c) assessment of sustainability can occur in services that transition earlier [67]. The design is particularly suited to implementation trials of service delivery interventions as it ensures all sites receive the intervention, while allowing assessment of whether (a) a change in quit rates has occurred; (b) the change is likely due to the intervention; and (c) the change is significant [49]. The 32-month timeframe of the MOHMQuit trial facilitates measurement of the success of the intervention over a long period, also enabling the sustainability of the intervention to be assessed. Finally, the stepped-wedge design also allowed for flexibility in adapting the design of the study in response to delays resulting from the impact of COVID-19 on the health care system and redesign work being completed in *e*Maternity.

The inclusion of both an economic evaluation and a process evaluation are further strengths of this trial, providing information on costs of implementation, cost-effectiveness, fidelity of delivery, and factors critical for effective implementation. Detailed protocols for each of these sub-studies will be published separately. As reducing smoking in pregnancy is an identified priority for NSW Health, this ‘real-world’ evidence will support decision-making by our policy partners for broader ‘at-scale’ implementation if appropriate.

### Demonstrating benefits of partnership projects and collaboration

The MOHMQuit intervention has the potential to demonstrate the benefits of partnership projects involving collaboration between policy-makers, clinicians and researchers from conception through implementation to address a population health problem [65, 68, 69]. In the research design phase, collaboration between all MOHMQuit partners helped place the policy and practice relevance of the research central to the research design [65, 68]. Ongoing collaboration with the midwifery and obstetric partner investigators at each research site will help ensure that the implementation of the intervention reflects real world practice and build the research capacity of the partner investigators [65, 68, 70, 71], increasing the likelihood of the findings being used, adopted and sustained beyond the trial timeframe. Our approach also supports broader scale up and implementation across the state, due to strong engagement of policy-makers at all stages from development of MOHMQuit to the trial itself, with potential relevance nationally and internationally.

Maternity care clinicians can play a crucial role in helping women stop smoking in pregnancy by providing evidence-based guideline-recommended SCS during antenatal care [15, 17, 23, 72]. Considering that stopping smoking in pregnancy significantly reduces the risk of adverse pregnancy outcomes, maternal-foetal health complications, ongoing developmental complications in the baby [1, 73], multiple complications including stillbirth, preterm birth and low birth weight and birth defects [1, 3], the potential benefit of achieving smoking cessation amongst pregnant women through the MOHMQuit intervention is considerable. MOHMQuit, if successful, will improve maternal and infant outcomes and has the potential for scale-up across the wider public health maternity care system in Australia.

## Supplementary Information

**Additional file 1.** MOHMQuit Behaviour Change Wheel barriers, intervention types, behaviour change techniques, outcomes and measures.

**Additional file 2.** Details of the training, resources and delivery of the MOHMQuit intervention.

**Additional file 3.** Completed Consolidated Standards of Reporting Trials (CONSORT) extension for the stepped wedge cluster randomised trial.

**Additional file 4.** Completed TIDieR (Template for Intervention Description and Replication) Checklist.

## Data Availability

Not applicable.

## Abbreviations

5As: 5As of smoking cessation support: Ask, Advise, Assess, Assist and Arrange follow-up
ACTRN12622000167763: MOHMQuit Trial registration number in the Australian New Zealand Clinical Trials Registry (ANZCTR)
CONSORT extension checklist: Consolidated Standards of Reporting Trials (CONSORT) extension for the stepped wedge cluster randomised trials
COVID-19: Viral disease caused by the novel coronavirus SARS-CoV2
HETI: The NSW Health Education and Training Institute (run through the NSW health system)
MOHMQuit Trial: The Midwives and Obstetricians Helping Mothers to Quit Smoking Trial
NRT: Nicotine replacement therapy
NSW: New South Wales
NSW Quitline: A confidential telephone information and advice service to help smokers quit smoking and remain abstinent
PIS: Participant Information Statement (may be given in paper or appear in electronic form as the first page of a questionnaire)
PRECIS-2 criteria: PRagmatic Explanatory Continuum Indicator Summary tool criteria - eligibility criteria, recruitment, setting, organisation, flexibility (delivery), flexibility (adherence), follow-up, primary outcome, and primary analysis.
QALY: Quality adjusted life year
REDCap: Research Electronic Data Capture application
SCS: Smoking cessation support
SSIs: Semi-structured interviews
TIDieR Checklist: Template for Intervention Description and Replication Checklist
USYD RDS: The University of Sydney’s Research Data Store
