# Supplementary material for: The MOHMQuit (Midwives and Obstetricians Helping Mothers to Quit Smoking) Trial: protocol for a stepped-wedge implementation trial to improve best practice smoking cessation support in public antenatal care services
Source: Implement Sci. 2022 Dec 9;17:79. doi: 10.1186/s13012-022-01250-3 (PMC9734467; doi:10.1186/s13012-022-01250-3)
Supplement: Supplementary file 1 — Additional file 1. MOHMQuit Behaviour Change Wheel barriers, intervention types, behaviour change techniques, outcomes and measures. [file 13012_2022_1250_MOESM1_ESM.pdf]

Additional File 1. MOHMQuit Behaviour Change Wheel[1] barriers, intervention types, behaviour change techniques, outcomes and measures.

Barrier	Intervention types	Behaviour Change Techniques	Measures – how we will assess whether we have addressed the Barrier?
**Clinicians**			
Poor knowledge, skills and confidence in providing smoking cessation support (SCS), especially with Assisting and Arranging follow-up, confusion about cutting down vs quitting	Education<br>Training<br>Persuasion<br>Incentivisation	Education<br>• 4.1 Instruction on how to perform a behaviour *(information on how and when to provide the 5As)*<br>• 6.3 Information about others approval (*explain Ministry of Health policy and guidelines*).<br>• 9.1 Credible source *(above information provided by credible source)*<br>• 5.1 Information about health consequences (*provide information on the risks/benefits of quitting vs cutting down*)<br><br>Training<br>• 4.1 Instruction on how to perform a behaviour *(detailed information on how to assist women with strategies including use of NRT in pregnancy; and how to motivate them)*<br>• 6.1 Demonstration of the behaviour *(demonstration of how to perform each of the elements of assisting under varying circumstances)*<br>• 8.1 Behavioural practice/ rehearsal *(practice performing the elements of assisting using role play)*<br><br>Persuasion<br>• 15.3 Focus on past success/ and 15.1 Verbal persuasion about capability *(highlight communication skills midwives have developed in other areas)*<br>• 9.1 Credible source *(delivered by senior or other respected midwife*)<br>Incentivisation<br>• 10.4 Social reward *(praise for practising behaviour during and after intervention training sessions)*	Our validated clinician questionnaire was designed based on the TDF.[2] Factor analysis identified nine barrier/enabler factors, including one named *Capability*.<br>This factor includes 12 items measuring self-perceived capability (knowledge, skills, confidence). Capability was found to be significantly associated with provision of SCS in our initial cross-sectional survey[3].<br><br>The items are included in the questionnaire with clinicians at three timepoints:<br>1. Immediately pre-training<br>2. Immediately post-training<br>3. 6 months after the training

Barrier	Intervention types	Behaviour Change Techniques	Measures – how we will assess whether we have addressed the Barrier?
SCS perceived as low priority relative to addressing other conditions/issues, and 5As took too long.	Training<br>Persuasion	Training<br>• 4.1 Instruction on how to perform a behaviour (*reiterating 5As designed to be delivered in a short consultation*)<br>• 6.1 Demonstration of the behaviour (*how to have 5As discussions whilst doing other clinical duties*)<br>• 8.1 Behavioural practice/ rehearsal (*practice performing the elements of assisting using role play*)<br><br>Persuasion<br>• 5.1 Information about health consequences/ and 5.2 Salience of consequences (*impact of smoking relative to another condition they manage well eg diabetes*)<br>• 9.3 Comparative imagining of future outcomes/and 13.2 Framing/reframing (*reframing action for smoking by comparing with action for other condition*)	In our validated clinician questionnaire three items load onto a factor named *Personal Priority*. Personal Priority was found to be significantly associated with provision of SCS in our initial cross-sectional survey.<br><br>The items are included in the questionnaire with clinicians at three timepoints:<br>1. Immediately pre-training<br>2. Immediately post-training<br>3. 6 months after the training
There were no mechanisms or systems for clinicians to use to monitor/self-monitor if they were following the 5As, and no systems for them to track smokers and the support provided	Education<br>Enablement<br>Environmental restructuring	Education<br>• 2.2 Feedback on the behaviour (*provide information on clinic performance providing SCS)*<br><br>Enablement<br>• 1.2 Problem solving/ and<br>• 1.4 Action planning/ and<br>• 2.3 Self-monitoring<br>(*encourage midwives to problem solve barriers and solutions to self-monitoring, and make a plan to manage this)*<br><br>Environmental restructuring<br>• 7.1 Prompts/cues (*modify EMR to include flags for smokers, build in reminders to follow 5As at every antenatal visit and ensure key fields are included for all antenatal visits)*	In our validated clinician questionnaire two items that load onto a factor named *Tracking systems*. Tracking systems was found to be significantly associated with provision of SCS in our initial cross-sectional survey.<br><br>The items are included in the questionnaire with clinicians at two timepoints:<br>1. Immediately pre-training<br>2. 6 months after the training

Barrier	Intervention types	Behaviour Change Techniques	Measures – how we will assess whether we have addressed the Barrier?
		• 12.5 Adding objects to the environment (*as above for 7.1; and summary guide of the 5As, assist and arrange follow-up flip book, helpful hints for clinicians, reference card to pin on badge holder)*	
Lack of champions at all levels including both managers and peers.	Enablement	Enablement<br>• 12.2 Restructuring the social environment *(manager encouraging attendance at training; resource for managers on developing and supporting champions; leadership training components below)*	In our validated clinician questionnaire five items load onto a factor named *Work environment*. One relates specifically to champions.<br>Both the Work environment factor and the individual items were found to be significantly associated with provision of SCS in our initial cross-sectional survey.<br><br>The items are included in the questionnaire with clinicians at two timepoints:<br>1. Immediately pre training<br>2. 6 months after the training
Lack of printed resources to use with women who smoke, or printed resources were out of date or not specific to pregnancy.	Environmental restructuring	Environmental restructuring<br>• 12.5 Adding objects to the environment *(all the various resources for clinicians, especially the self-help booklet for use with women, NRT information sheet for women)*	One of the items in the *Work environment* factor (see above) is specific to availability of resources.<br><br>The items are included in the questionnaire with clinicians at two timepoints:<br>1. Immediately pre-training<br>2. 6 months after the training
Some did not consider referral to Quitline to be effective.	Education<br>Persuasion	Education<br>• 5.1 Information about health consequences *(that referrals to Quitline result in x% increase in quit attempts/rates)*<br><br>Persuasion<br>9.1 Credible source (*delivered by Quitline staff or other cessation expert)*	One item in the clinician questionnaire measures participants' perceptions of the effectiveness of referral to Quitline in helping women quit. It didn't load onto a factor but the single item was significantly associated with self-reported: referral to Quitline; provision of self-help materials; and scheduling follow-up in our initial survey.

Barrier	Intervention types	Behaviour Change Techniques	Measures – how we will assess whether we have addressed the Barrier?
			It will be administered with clinicians at three timepoints:<br>1. Immediately pre-training<br>2. Immediately post-training<br>3. 6 months after the training
Some midwives have concerns about damaging the client relationship.	Persuasion<br>Modelling	Modelling<br>• 6.1 Demonstration of the behaviour *(video showing engaged client and effective midwife)*<br><br>Persuasion<br>• 5.1 Information about health consequences/ and 5.3 Information about social and environmental consequences/ and 6.3 Information about others approval *(professional patient describing health and emotional (not valued) consequences of midwife not addressing their smoking – gives impression OK to keep smoking)*<br>• 9.1 Credible source (*above information delivered by a pregnant or postpartum woman who smoked)*	One additional item was added to the questionnaire to assess this issue (previously identified through qualitative data, not the questionnaire).<br><br>It will be administered at three timepoints:<br>1. Immediately pre-training<br>2. Immediately post-training<br>3. 6 months after the training
Framing smoking as a social issue/lifestyle choice rather than an addiction (and therefore not my role).	Education<br>Persuasion	Education<br>• 5.1 Information about health consequences *(Impact of nicotine on the brain and role in addiction)*<br><br>• Persuasion<br>• 9.3 Comparative imagining of future outcomes *(comparison with their successful responses to other behavioural issues e.g., domestic violence)*<br>• 13.2 Framing/reframing *(reframing smoking as a behavioural indicator for intervention rather than a 'lifestyle choice')*	One additional item was added to the questionnaire to assess this issue (previously identified through qualitative data, not the questionnaire).<br><br>It will be administered at three timepoints:<br>1. Immediately pre-training<br>2. Immediately post-training<br>3. 6 months after the training
Some midwives uncomfortable asking about smoking.	Persuasion<br>Modelling	Modelling<br>• 6.1 Demonstration of the behaviour *(video showing engaged client and effective midwife)*<br><br>Persuasion	One additional item was added to the questionnaire to assess this issue (previously identified through qualitative data, not the questionnaire).<br><br>It will be administered at three timepoints:<br>1. Immediately pre-training

Barrier	Intervention types	Behaviour Change Techniques	Measures – how we will assess whether we have addressed the Barrier?
		• 5.1 Information about health consequences/ and 5.4 Information about social and environmental consequences *(professional patient describing health and emotional (not valued) consequences of midwife not addressing their smoking)*<br>• 9.1 Credible source *(professional patient)*<br>• 5.4 Information about social and environmental consequences *(other midwives feel good about professional role after delivering 5As)*<br>• 9.1 Credible source *(midwifery champion)*	2. Immediately post-training<br>3. 6 months after the training
**Leaders**			
Lack of leadership for smoking cessation	Environmental restructuring<br>Training<br>Enablement<br>Persuasion	Environmental restructuring<br>• 12.5 Adding objects to the environment *(clinic action planning tool; guidance on developing and supporting champions; guidance on developing local care pathways for SCS)*<br>Training<br>• 4.1 Instruction on how to perform a behaviour *(instruction in use of resources above)*<br>• 6.1 Demonstration of the behaviour *(demonstration of how to commence action planning and care pathways)*<br>Enablement<br>• 1.2 Problem solving and 1.4 Action planning *(leaders problem solving issues and planning for use of resources, during training)*<br>• 12.2 Restructuring the social environment *(encouraging formation of 'community of practice' among leaders of participating services)*<br>Persuasion<br>• 5.1 Information about health consequences/ and 5.2 Salience of consequences (*impact of smoking relative to another condition they manage well e.g., diabetes*)<br>• 9.3 Comparative imagining of future outcomes/and 13.2 Framing/reframing (*reframing action for smoking by comparing with action for other condition*)	A questionnaire for leaders administered 3 months after training includes self-assessment of leadership using the *Implementation Leadership Scale* (for supervisors).[4]<br><br>The four item *Leadership Engagement Scale*[5] will be included in the clinician questionnaire administered at 6 months post-training<br><br>In our validated clinician questionnaire five items load onto a factor named *Work environment*. Two items in the Work Environment factor relate to leadership:<br><br>The items are included in the questionnaire with clinicians at two timepoints:<br>1. Immediately before the training<br>2. 6 months after the training<br><br>Qualitative interviews with a lynchpin leader (as identified by the clinical midwifery consultant

Barrier	Intervention types	Behaviour Change Techniques	Measures – how we will assess whether we have addressed the Barrier?
			[CMC]), the CMC and the lead obstetrician in each site will explore leadership issues
There was no system to monitor smoking cessation support that women received, for quality improvement purposes.	Environmental restructuring<br>Training	Environmental restructuring<br>• 12.5 Adding objects to the environment (*develop a reporting system for managers to monitor cessation support provided)*<br><br>Training<br>• 4.1 Instruction on how to perform a behaviour *(train key leaders in use of the reporting system and its use for QI)*	One item in the questionnaire for leaders at 3 months asks about using the eMaternity reports for QI purposes<br><br>Qualitative interviews with a lynchpin leader (as identified by the CMC), the CMC and the lead obstetrician in each site will explore this issue.

Key:* 5As** *= 5As of smoking cessation support: Ask, Advise, Assess, Assist and Arrange follow-up;* ***CMC *= clinical midwifery consultant;* ***eMaternity*** *= the electronic health record used by maternity services in New South Wales;* ***QI*** *= quality improvement;* ***Quitline*** *= a state-based telephone Quitline service in New South Wales, Australia;* ***SCS*** *= smoking cessation support;* ***TDF*** *= Theoretical Domains Framework[2], a framework of psychological constructs in behaviour change theory – to identify barriers and enablers to behaviour change.*

References for Additional File 1

1. Michie S, Atkins L, West R. The Behaviour Change Wheel - A Guide to Designing Interventions. United Kingdom: Silverback Publishing; 2014.
2. Cane J, O'Connor D, Michie S. Validation of the theoretical domains framework for use in behaviour change and implementation research. Implement Sci. 2012;7:37, https://doi.org/10.1186/1748-5908-7-37.
3. Passey ME, Longman JM, Adams CM, Johnston JJ, Simms J, Rolfe MI. Factors associated with provision of smoking cessation support to pregnant women – a cross-sectional survey of midwives in New South Wales, Australia. BMC Pregnancy and Childbirth. 2020;20:219, https://doi.org/10.1186/s12884-020-02912-0.
4. Aarons GA, Ehrhart MG, Farahnak LR. The Implementation Leadership Scale (ILS): Development of a Brief Measure of Unit Level Implementation Leadership. Implement Sci. 2014;9:45. doi:10.1186/1748-5908-9-45.
5. Fernandez ME, Walker TJ, Weiner BJ, Calo WA, Liang S, Risendal B, et al. Developing measures to assess constructs from the Inner Setting domain of the Consolidated Framework for Implementation Research. Implement Sci. 2018;13(1):52. doi:10.1186/s13012-018-0736-7.
