# Supplementary material for: The MOHMQuit (Midwives and Obstetricians Helping Mothers to Quit Smoking) Trial: protocol for a stepped-wedge implementation trial to improve best practice smoking cessation support in public antenatal care services
Source: Implement Sci. 2022 Dec 9;17:79. doi: 10.1186/s13012-022-01250-3 (PMC9734467; doi:10.1186/s13012-022-01250-3)
Supplement: Supplementary file 2 — Additional file 2. Details of the training, resources and delivery of the MOHMQuit intervention. [file 13012_2022_1250_MOESM2_ESM.pdf]

Additional File 2. Details of the training, resources and delivery of the MOHMQuit Intervention

Table A2.2 Details of the training, resources and delivery of the MOHMQuit Intervention

Target group	Activities^	Resources
**Maternity service leaders, midwives, Aboriginal health workers, obstetricians and obstetric trainees**	**Two 30-minute online training modules from HETI^^ covering:**<br>(i) Harms of antenatal smoking<br>(ii) The evidence for SCS<br>(iii) Use of the 5As<br>(iv) Use of nicotine replacement therapy (NRT) in pregnancy<br>*Note - it is intended that participants will have completed the HETI modules to ensure basic knowledge prior to MOHMQuit-specific training, which reinforces the learning presented in the modules.*	*Not applicable*
**Maternity service leadership group:**<br>• Midwifery unit managers<br>• Clinical midwifery consultants<br>• Clinical midwifery educators<br>• Other senior midwives	**3-hour workshop covering:**<br>• SCS leadership<br>• Reviewing eMaternity* reports (local performance data on provision of SCS)<br>• Action planning<br>• Developing care pathways for SCS<br>• Developing and maintaining champions	• Template for generating eMaternity reports on provision of SCS in their clinic<br>• Guidance on use of the eMaternity reports for quality improvement<br>• Comparison with action for other conditions e.g., gestational diabetes<br>• A clinic/service action planning tool<br>• Guidance on developing champions<br>• Guidance on developing local care pathways<br>• Summary of evidence of effectiveness of 5As, Quitline and interventions in pregnancy

**Clinical midwifery educators**<br>• In addition to attending the leadership workshop above, and attending the clinician training, educators at each site will attend an additional training to maximise sustainability of the intervention.	**1-hour workshop covering:**<br>• How to provide MOHMQuit training to midwives, Aboriginal health workers and obstetricians.	• A specific train-the-trainer resource pack that includes all the materials used in the training for midwives, Aboriginal health workers and obstetricians<br>• Instruction on how to run training<br>• Guidance on adapting the MOHMQuit training to different situations (e.g., one-on-one support, half-hour brief team training, or the full one-day workshop)
**Midwives and Aboriginal health workers:**<br>• All midwives providing antenatal care in any setting<br>• All Aboriginal health workers providing antenatal care in any setting<br>*N.B. settings may include hospital or community-based clinics, outreach programs, and hospital wards*	**Full-day workshop covering:**<br>• Importance of providing SCS<br>• How to provide effective SCS<br>• How to use the MOHMQuit resources<br>• Using tools for self-monitoring provision of SCS and action planning<br>• Documentation of SCS in eMaternity to accurately record SCS provided in eMaternity, to enhance record keeping and communication with other clinicians	• 11 short videos demonstrating critical techniques in providing SCS<br>• Guidance on recording smoking information in eMaternity<br>• Information on NSW Quitline**<br>• Comparison with action for other conditions e.g., gestational diabetes<br>• Summary guide of the 5As<br>• Assist and arrange follow-up flip booklet<br>• Helpful hints for clinicians<br>• Reference card to attach to ID card<br>• Self-help booklet for use with women<br>• NRT (nicotine replacement therapy) information sheets for clinicians<br>• NRT information for women

**Obstetricians and obstetric trainees involved in antenatal care**	**2-hour training covering:**<br>• Importance of providing SCS<br>• How to provide effective SCS<br>• Use of the MOHMQuit resources<br>• Documentation of SCS in eMaternity	• 11 short videos demonstrating critical techniques in providing SCS<br>• Guidance on recording smoking information in eMaternity<br>• Information on NSW Quitline**<br>• Comparison with action for other conditions e.g., gestational diabetes<br>• Summary guide of the 5As<br>• Assist and arrange follow-up flip booklet<br>• Helpful hints for clinicians<br>• Reference card to attach to ID card<br>• Self-help booklet for use with women<br>• NRT (nicotine replacement therapy) information sheets for clinicians<br>• NRT information for women
**Maternity service leaders, midwives, Aboriginal health workers, obstetricians and obstetric trainees who have attended the MOHMQuit Training.**<br>*Attendees will be invited to attend at their discretion.*	**Community of Practice meetings (1-hour meetings, held online)**<br>• Five weeks after training occurs at their site, each site will be encouraged to participate in Community of practice meetings, facilitated online by the research team.<br>• Meetings will continue monthly and provide additional and ongoing support to all leaders and clinicians and encourage peer support and encouragement.	*Not applicable*

^While it is intended to run both the leader and clinician workshops in a face-to-face format, remote options (video-conferencing etc) will be used if required because of COVID-19 restrictions. Remote options will also be used to supplement or reinforce the face-to-face training, as required, including the Community of Practice meetings.
^^HETI (The NSW Health Education and Training Institute) provides training and education to support clinicians, trainers, managers and leaders across the NSW health system. The Institute has developed two 30-minute online training modules on supporting pregnant women to quit smoking.
*eMaternity is the electronic health record used by maternity services in New South Wales.
**NSW Quitline is confidential telephone information and advice service to help smokers quit smoking and remain abstinent.
