# Supplementary material for: The MOHMQuit (Midwives and Obstetricians Helping Mothers to Quit Smoking) Trial: protocol for a stepped-wedge implementation trial to improve best practice smoking cessation support in public antenatal care services
Source: Implement Sci. 2022 Dec 9;17:79. doi: 10.1186/s13012-022-01250-3 (PMC9734467; doi:10.1186/s13012-022-01250-3)
Supplement: Supplementary file 3 — Additional file 3. Completed Consolidated Standards of Reporting Trials (CONSORT) extension for the stepped wedge cluster randomised trial. [file 13012_2022_1250_MOESM3_ESM.pdf]

Additional File 3. Completed Consolidated Standards of Reporting Trials (CONSORT) extension for the stepped wedge cluster randomised trial

Table A3. CONSORT 2010 checklist of information to include when reporting a cluster randomised trial.(1)

Section/Topic	Item No	Checklist item	Section of the paper
	1a	Identification as a cluster randomised trial in the title.	Title page
	1b	Structured summary of trial design, methods, results, and conclusions (see separate SW-CRT checklist for abstracts).	Abstract (partly applicable only – protocol). Abstract includes Background, Methods, Discussion and Trial registration.
Background and objectives	2a	Scientific background. Rationale for using a cluster design.	Methods, study design
	2b	Specific objectives or hypotheses.	- Primary hypothesis: Methods<br>- Aims: Study design<br>- Outcomes: Methods, Data collection and analysis, Primary outcome (health outcome) and Table 2 details the secondary health outcome, intervention outcomes, moderators, mediators and economic evaluation.
Trial design	3a	Description and diagram of trial design including definition of cluster, number of sequences, number of clusters randomised to each sequence, number of periods, duration of time between each step, and whether the participants assessed in different periods are the same people, different people, or a mixture.	- Description and diagram of trial design with all details: Methods, Study design.<br>- Note: Figure 2 in the Methods illustrates the Implementation of MOHMQuit and the stepped-wedge cluster-randomised trial design.
	3b	Important changes to methods after trial commencement (such as eligibility criteria), with reasons.	Not applicable (protocol paper)
Participants	4a	Eligibility criteria for clusters and participants.	- Eligibility for clusters (sites): Methods, Participants, eligibility – study sites<br>- Eligibility for participants (maternity service leaders and clinicians): Methods, Participants, eligibility – maternity service leaders and clinicians

			- Eligibility for participants (pregnant and postpartum women): Methods, Participants, eligibility – pregnant and postpartum women
	4b	Settings and locations where the data were collected.	Methods, Settings
Interventions	5	The intervention and control conditions with sufficient details to allow replication, including whether the intervention was maintained or repeated, and whether it was delivered at the cluster level, the individual participant level, or both.	- Methods, The implementation intervention - MOHMQuit<br>- Methods, Study design
Outcomes	6a	Completely defined pre-specified primary and secondary outcome measures, including how and when they were assessed.	- Methods, Primary hypothesis<br>- Outcomes: Methods, Data collection and analysis, Primary outcome (health outcome) and Table 2 details the secondary health outcome, intervention outcomes, moderators, mediators and economic evaluation<br>- Note: Table 2 and Figure 3 also provide information on primary and secondary outcome measures, sources of data and timepoints of collection.
	6b	Any changes to trial outcomes after the trial commenced, with reasons.	Not applicable (protocol paper)
Sample size	7a	How sample size was determined. Method of calculation and relevant parameters with sufficient detail so the calculation can be replicated. Assumptions made about correlations between outcomes of participants from the same cluster. (See separate checklist for SW-CRT sample size items).	Methods, Sample size
	7b	When applicable, explanation of any interim analyses and stopping guidelines.	Not applicable (protocol paper)
Sequence generation	8a	Method used to generate the random allocation sequence.	Methods, Randomisation and blinding
	8b	Type of randomisation; details of any constrained randomisation or stratification, if used.	Not applicable (protocol paper)

Allocation concealment mechanism	9	Specification that allocation was based on clusters; description of any methods used to conceal the allocation from the clusters until after recruitment.	Methods, Randomisation and blinding
Implementation	10a	Who generated the randomisation schedule, who enrolled clusters, and who assigned clusters to sequences.	- Randomisation schedule: Methods, Randomisation and blinding<br>- Enrolment of clusters/services: Methods, Recruitment and consent – Services; and Methods, Eligibility criteria – Study sites<br>- Who assigned clusters to sequences: Methods, Randomisation and blinding
	10b	Mechanism by which individual participants were included in clusters for the purposes of the trial (such as complete enumeration, random sampling; continuous recruitment or ascertainment; or recruitment at a fixed point in time), including who recruited or identified participants.	- Methods, Recruitment and consent, Staff (maternity service leaders and clinicians)<br>- Methods, Recruitment and consent, Pregnant and postpartum women
	10c	Whether, from whom and when consent was sought and for what; whether this differed between treatment conditions.	- Methods, Recruitment and consent, Staff (maternity service leaders and clinicians)<br>- Methods, Recruitment and consent, Pregnant and postpartum women
Blinding	11a	If done, who was blinded after assignment to sequences (e.g., cluster level participants, individual level participants, those assessing outcomes) and how.	Methods, Randomisation and blinding
	11b	If relevant, description of the similarity of interventions.	Not relevant
Statistical methods	12a	Statistical methods used to compare treatment conditions for primary and secondary outcomes including how time effects, clustering and repeated measures were taken into account.	Clustering and repeated measures:<br>- Methods, Data collection and analysis, Primary outcome (health outcome)<br>- Methods, Data collection and analysis, Table 2
	12b	Methods for additional analyses, such as subgroup analyses, sensitivity analyses, and adjusted analyses.	Sensitivity analyses:<br>- Methods, Data collection and analysis, Table 2
Participant flow (a diagram is strongly recommended)	13a	For each treatment condition or allocated sequence, the numbers of clusters and participants who were assessed for eligibility, were randomly assigned, received intended treatments, and were	Not applicable (protocol paper)

		analysed for the primary outcome (see separate SW-CRT flow chart).	
	13b	For each treatment condition or allocated sequence, losses and exclusions for both clusters and participants with reasons.	Not applicable (protocol paper)
Recruitment	14a	Dates defining the steps, initiation of intervention, and deviations from planned dates. Dates defining recruitment and follow-up for participants.	Not applicable (protocol paper)
	14b	Why the trial ended or was stopped.	Not applicable (protocol paper)
Baseline data	15	Baseline characteristics for the individual and cluster levels as applicable for each treatment condition or allocated sequence.	Not applicable (protocol paper)
Numbers analysed	16	The number of observations and clusters included in each analysis for each treatment condition and whether the analysis was according to the allocated schedule.	Not applicable (protocol paper)
Outcomes and estimation	17a	For each primary and secondary outcome, results for each treatment condition, and the estimated effect size and its precision (such as 95% confidence interval); any correlations (or covariances) and time effects estimated in the analysis.	Not applicable (protocol paper)
	17b	For binary outcomes, presentation of both absolute and relative effect sizes is recommended.	Not applicable (protocol paper)
Ancillary analyses	18	Results of any other analyses performed, including subgroup analyses and adjusted analyses, distinguishing pre-specified from exploratory.	Not applicable (protocol paper)
Harms	19	All important harms or unintended effects in each group (for specific guidance see CONSORT for harms).	Not applicable (protocol paper)
Limitations	20	Trial limitations, addressing sources of potential bias, imprecision, and, if relevant, multiplicity of analyses.	Not applicable (protocol paper)
Generalisability	21	Generalisability (external validity, applicability) of the trial findings. Generalisability to clusters and/or individual participants (as relevant).	Not applicable (protocol paper)
Interpretation	22	Interpretation consistent with results, balancing benefits and harms, and considering other relevant evidence.	Not applicable (protocol paper)

Registration	23	Registration number and name of trial registry.	Abstract
Protocol	24	Where the full trial protocol can be accessed, if available.	Not applicable (protocol paper)
Funding	25	Sources of funding and other support (such as supply of drugs), and the role of funders.	Declarations, Funding
Research ethics review	26	Whether the study was approved by a research ethics committee, with identification of the review committee(s). Justification for any waiver or modification of informed consent requirements.	Declarations, Ethics approval and consent to participate

Reference for Additional File 3

1. Hemming K, Taljaard M, McKenzie JE, Hooper R, Copas A, Thompson JA, et al. Reporting of stepped wedge cluster randomised trials: extension of the CONSORT 2010 statement with explanation and elaboration. BMJ. 2018;363.
