# Supplementary material for: The MOHMQuit (Midwives and Obstetricians Helping Mothers to Quit Smoking) Trial: protocol for a stepped-wedge implementation trial to improve best practice smoking cessation support in public antenatal care services
Source: Implement Sci. 2022 Dec 9;17:79. doi: 10.1186/s13012-022-01250-3 (PMC9734467; doi:10.1186/s13012-022-01250-3)
Supplement: Supplementary file 4 — Additional file 4. Completed TIDieR (Template for Intervention Description and Replication) Checklist. [file 13012_2022_1250_MOESM4_ESM.pdf]

Additional File 4. Completed TIDieR (Template for Intervention Description and Replication) Checklist

Hoffmann et al. (1) state that the focus of the TIDieR Checklist is to report "details of the intervention elements (and where relevant, comparison elements) of a study. Other elements and methodological features of studies are covered by other reporting statements and checklists and have not been duplicated as part of the TIDieR checklist". The TIDieR Checklist for the paper '*The MOHMQuit (Midwives and Obstetricians Helping Mothers to Quit Smoking) Trial: protocol for a stepped-wedge implementation trial to improve best practice smoking cessation support in public antenatal care services*' has been completed in conjunction with the CONSORT 2010 checklist of information to include when reporting a cluster randomised trial (2).

Table A4.1. The TIDieR (Template for Intervention Description and Replication) Checklist - Information to include when describing an intervention and the location of the information.(1, 3)

**Item number**	**Item**	**Details**	**Where located (section/s of the primary paper or Additional file, or other details)**
	**BRIEF NAME**		
**1.**	Provide the name or a phrase that describes the intervention.	The MOHMQuit (Midwives and Obstetricians Helping Mothers to Quit) intervention	Title<br>Methods, The implementation intervention - MOHMQuit
	**WHY**		
**2.**	Describe any rationale, theory, or goal of the elements essential to the intervention.	The MOHMQuit intervention was developed using the Behaviour Change Wheel method in order to improve implementation of guidelines for supporting smoking cessation during pregnancy, among antenatal care providers working in the public health system.	Abstract, Background<br><br>Methods, The implementation intervention - MOHMQuit<br><br>Additional File 1. MOHMQuit Behaviour Change Wheel Intervention types and Behaviour Change Techniques to address identified barriers and support identified enablers
	**WHAT**		
**3.**	Materials: Describe any physical or informational materials used in the intervention, including those provided to participants or used in intervention delivery or in training of intervention providers. Provide information on where the	The MOHMQuit intervention includes the following materials:<br><u>For leaders</u> (midwifery unit managers, clinical midwifery consultants, clinical midwifery educators, other senior midwives):<br>• Template for generating *e*Maternity^ reports on provision of SCS in their clinic<br>• Guidance on use of the *e*Maternity^ reports for quality improvement<br>• Comparison with action for other conditions e.g., gestational hypertension<br>• A clinic/service action planning tool	Additional File 1. MOHMQuit Behaviour Change Wheel Intervention types and Behaviour Change Techniques to address identified barriers and support identified enablers

	materials can be accessed (e.g. online appendix, URL).	• Guidance on developing champions<br>• Guidance on developing local care pathways<br><br><u>For Clinical midwifery educators</u><br>• A specific train-the-trainer resource pack that includes all the materials used in the training for midwives, Aboriginal health workers and obstetricians<br>• Instruction on how to run training<br>• Guidance on adapting the MOHMQuit training to different situations (e.g., one-on-one support, half-hour brief team training, or the full one-day workshop)<br><br><u>For clinicians</u> (midwives, Aboriginal Health Workers and obstetricians):<br>• 11 short videos demonstrating critical techniques in providing SCS<br>• Guidance on recording smoking information in *e*Maternity^<br>• Information on NSW Quitline^^<br>• Comparison with action for other conditions e.g, gestational hypertension<br>• Summary guide of the 5As<br>• Assist and arrange follow-up flip booklet<br>• Helpful hints for clinicians<br>• Reference card to attach to ID card<br>• Self-help booklet for use with women<br>• NRT information sheets for clinicians<br>• NRT information for women	Additional File 2. Details of the training, resources and delivery of the MOHMQuit intervention<br><br>*MOHMQuit materials are not currently publicly available at the time of publication of this protocol paper, as the MOHMQuit Intervention is being trialled.*
**4.**	Procedures: Describe each of the procedures, activities, and/or processes used in the intervention, including any enabling or support activities.	The MOHMQuit intervention includes several stages:<br><u>All participants</u> will be requested to complete two short online training modules developed by the NSW Health Education and Training Institute (HETI), prior to other components. These modules address:<br>• NSW Health policy regarding SCS<br>• Knowledge of harms of smoking in pregnancy<br>• The evidence for SCS<br>• Use of the 5As<br>• Use of NRT in pregnancy<br><br><u>Clinical Midwifery Consultants</u> (CMCs):<br>• Initial engagement to clarify their role in supporting practice change and the MOHMQuit intervention<br><br><u>For leaders</u>:	Methods, The implementation intervention – MOHMQuit, *Maternity service leaders workshop, Clinician workshops, Clinical midwifery educator training, Development of a Community of Practice*<br><br>Additional File 2. Details of the training, resources and delivery of the MOHMQuit intervention

		3-hour workshop covering:<br>• SCS leadership<br>• Reviewing *e*Maternity^ reports (local performance data on provision of SCS)<br>• Action planning, including initial steps<br>• Developing care pathways for SCS<br>• Developing and maintaining champions<br>• Expectations regarding next steps and support for MOHMQuit within their service, including development of a plan with deadlines for actions<br><br><u>For midwives and Aboriginal Health Workers</u>:<br>Full-day workshop covering:<br>• Importance of providing SCS<br>• How to provide effective SCS<br>• How to use the MOHMQuit resources<br>• Using tools for self-monitoring provision of SCS and action planning<br>• Documentation of SCS in *e*Maternity^<br><br><u>For obstetricians and obstetric trainees</u>:<br>• Importance of providing SCS<br>• How to provide effective SCS<br>• How to use the MOHMQuit resources<br>• Documentation of SCS in *e*Maternity^<br><br><u>Maternity service leaders, midwives, Aboriginal health workers, obstetricians and obstetric trainees who have attended the MOHMQuit Training:</u> Additionally, services will be encouraged to form a 'Community of practice' to provide additional and ongoing peer support and encouragement.<br>Clinical Midwifery Educators will be provided training and resources to continue to deliver training.	
	**WHO PROVIDED**		
**5.**	For each category of intervention provider (e.g., psychologist, nursing assistant), describe their expertise, background and any specific training given.	Training for the leadership group will be provided by a senior Clinical midwifery consultant (CMC) with extensive experience in leadership, evidence-based practice and training within NSW maternity services, and with detailed knowledge of all components of the MOHMQuit intervention.<br>Training for the clinical groups will be provided jointly by the CMC, and an accredited smoking cessation trainer, with experience specifically related to smoking in pregnancy.	Methods, The implementation intervention – MOHMQuit, *Maternity service leader workshop*<br><br>Methods, The implementation intervention – MOHMQuit, *Clinician workshops*

			Additional File 2. Details of the training, resources and delivery of the MOHMQuit intervention

	**HOW**		
**6.**	Describe the modes of delivery (e.g. face-to-face or by some other mechanism, such as internet or telephone) of the intervention and whether it was provided individually or in a group.	The HETI modules are provided on-line and it is intended that all participants will complete these in their own time before other components.<br><br>All MOHMQuit-specific training sessions are intended to be provided as face-to-face group training sessions. However, this may need to be revised in light of the COVID-19 pandemic and on-line or hybrid models will be considered, depending on circumstances.<br><br>Regardless of mode of delivery, all participants will be provided with the relevant resources for use in their services.<br><br>All clinicians (midwives, Aboriginal Health Workers, obstetricians and obstetric trainees) providing antenatal care will be asked to participate in the relevant training, and training will be offered several times at each site to maximise opportunity for participation.<br><br>Monthly Community of practice meetings will be facilitated online by the research team for all clinicians and leaders who have attended the MOHMQuit training. These Community of practice meetings will provide additional and ongoing support to all leaders and clinicians and encourage peer support and encouragement.	Methods, The implementation intervention – MOHMQuit, *Maternity service leaders workshop, Clinician workshops, Clinical midwifery educator training, Development of a Community of Practice*<br><br>Additional File 2. Details of the training, resources and delivery of the MOHMQuit intervention
	**WHERE**		
**7.**	Describe the type(s) of location(s) where the intervention occurred, including any necessary infrastructure or relevant features.	The MOHMQuit intervention will be provided at nine public maternity services in NSW. The sites vary in size and are located in rural and urban contexts. To be eligible for participation, maternity services were required to provide antenatal care and birthing services and to also (i) have a prevalence of smoking in the first half of pregnancy ≥12%; and (ii) have a minimum of 80 women smoking in the first half of pregnancy per year. These criteria ensured that the intervention will be offered in services with smoking prevalence higher than the Australian average of 9.5% in the first half of pregnancy; and provide an adequate number of smokers at each site. All	Methods, Settings<br><br>Methods, Eligibility criteria – Study sites

		antenatal services providing care to women intending to give birth at these sites were eligible to participate, including hospital based, community-based and outreach services.	
	**WHEN and HOW MUCH**		
**8.**	Describe the number of times the intervention was delivered and over what period of time including the number of sessions, their schedule, and their duration, intensity or dose.	The MOHMQuit intervention will be delivered in each participating site, with one session for the leadership group (3-hour workshop) and 2-3 sessions available (full day workshops) for midwives and Aboriginal Health Workers. The training for obstetricians and trainees (2-3 hour workshops) will be provided twice at each site. Additionally, to address the need to address staff turnover, the Clinical Midwifery Educators will be trained to provide ongoing training using a train-the-trainer model (1 hour workshop). Monthly Community of practice meetings (one-hour meetings) will be facilitated online by the research team for all clinicians and leaders who have attended the MOHMQuit training.	Methods, The implementation intervention – MOHMQuit, *Maternity service leaders workshop, Clinician workshops, Clinical midwifery educator training, Development of a Community of Practice*<br><br>Additional File 2. Details of the training, resources and delivery of the MOHMQuit intervention
	**TAILORING**		
**9.**	If the intervention was planned to be personalised, titrated or adapted, then describe what, why, when, and how.	Several elements of the intervention can be adapted to local services including action planning and developing care pathways for SCS, which will be developed by the local services. They will also be encouraged to review these annually.	Methods, The implementation intervention – MOHMQuit, *Maternity service leaders workshop, Clinician workshops, Clinical midwifery educator training, Development of a Community of Practice*<br><br>Additional File 2. Details of the training, resources and delivery of the MOHMQuit intervention
	**MODIFICATIONS**		
**10.‡**	If the intervention was modified during the course of the study, describe the changes (what, why, when, and how).	The intervention was modified following a small feasibility study (3), with additional enablement added to support the leadership group. This included engagement with CMCs in each participating site prior to commencement, to clarify expectations regarding their roles in supporting practice change and implementation of evidence-based care; development of a 'community of practice', once sites have received their initial training; greater clarity in the leadership training regarding expectations and next steps, with support to commence the action planning; and assistance for leaders in developing a 'roadmap' to plan next steps and deadlines for actions. Any subsequent changes during the larger implementation trial will be reported.	Reference (3): Passey ME, Adams C, Paul C, Atkins L, Longman JM. *Improving implementation of smoking cessation guidelines in pregnancy care: Development of an intervention to address system, maternity service leader and clinician factors*. Implementation Science Communications. 2021;2:128

		The trial was originally planned to run over 36 months at eight sites. However, due to the impacts of COVID-19 and some redesign work being completed in *e*Maternity, initiation of data collection and provision of the intervention was delayed. To compensate for the reduced time available for data collection, an additional site was added (site six in Figure 1), and the timing of providing the intervention at the final site brought forward. This ensured the study was still adequately powered.	Methods, Stepped-wedge cluster-randomised controlled trial design
	**HOW WELL**		
**11.**	Planned: If intervention adherence or fidelity was assessed, describe how and by whom, and if any strategies were used to maintain or improve fidelity, describe them.	This will be assessed in the larger trial in the several ways. The process evaluation will use data from questionnaires with midwives and obstetricians and Aboriginal health workers at six months post-intervention (secondary outcome 16b) and the questionnaire with leaders three months post-intervention (secondary outcomes 16a, 17, 18). In addition, semi-structured interviews with maternity service leaders at each site six months after the intervention will explore leaders' perceptions of acceptability, appropriateness, feasibility of implementation, adaptations and sustainability of the MOHMQuit intervention (secondary outcome 18).	Methods, Data collection and analysis, Table 2. MOHMQuit data collection and analysis
**12.**	Actual: If intervention adherence or fidelity was assessed, describe the extent to which the intervention was delivered as planned.	Not applicable. Not yet implemented.	*This item is not relevant to the protocol and cannot be described until the study is complete.*

^*e*Maternity is the electronic health record used by maternity services in New South Wales.

^^NSW Quitline is confidential telephone information and advice service to help smokers quit smoking and remain abstinent.

Key: *SCS = smoking cessation support; HETI = The New South Wales Health Education and Training Institute; 5As = 5As of smoking cessation support: Ask, Advise, Assess, Assist and Arrange follow-up; NRT = nicotine replacement therapy; NSW = New South Wales Australia; COVID-19 = viral disease caused by the novel coronavirus SARS-CoV2.*

References for Additional File 4.

1. Hoffmann TC, Glasziou PP, Boutron I, Milne R, Perera R, Moher D, et al. Better reporting of interventions: template for intervention description and replication (TIDieR) checklist and guide. BMJ. 2014;348:g1687.
2. Hemming K, Taljaard M, McKenzie JE, Hooper R, Copas A, Thompson JA, et al. Reporting of stepped wedge cluster randomised trials: extension of the CONSORT 2010 statement with explanation and elaboration. BMJ. 2018;363.
3. Passey ME, Adams C, Paul C, Atkins L, Longman JM. Improving implementation of smoking cessation guidelines in pregnancy care: Development of an intervention to address system, maternity service leader and clinician factors. Implementation Science Communications. 2021;2:128.
